# Circulating *inflamma-miRs* in aging and age-related diseases

**DOI:** 10.3389/fgene.2013.00121

**Published:** 2013-06-26

**Authors:** Fabiola Olivieri, Maria R. Rippo, Antonio D. Procopio, Francesca Fazioli

**Affiliations:** ^1^Department of Clinical and Molecular Sciences, Università Politecnica delle MarcheAncona, Italy; ^2^Center of Clinical Pathology and Innovative Therapy, I.N.R.C.A. National InstituteAncona, Italy

**Keywords:** inflammation mediators, circulating miRs, age-related diseases, NF-κB signaling, cellular senescence

## Abstract

Evidence on circulating microRNAs (miRNAs) is indisputably opening a new era in systemic and tissue-specific biomarker research, highlighting new inter-cellular and inter-organ communication mechanisms. Circulating miRNAs might be active messengers eliciting a systemic response as well as non-specific “by-products” of cell activity and even of cell death; in either case they have the potential to be clinically relevant biomarkers for a number of physiopathological processes, including inflammatory responses and inflammation-related conditions. A large amount of evidence indicates that miRNAs can exert two opposite roles, activating as well as inhibiting inflammatory pathways. The inhibitory action probably relates to the need for activating anti-inflammatory mechanisms to counter potent proinflammatory signals, like the nuclear factor kappaB (NF-κB) pathway, to prevent cell and tissue destruction. MiRNA-based anti-inflammatory mechanisms may acquire a crucial role during aging, where a chronic, low-level proinflammatory status is likely sustained by the cell senescence secretome and by progressive activation of immune cells over time. This process entails age-related changes, especially in extremely old age, in those circulating miRNAs that are capable of modulating the inflammatory status (*inflamma-miRs*). Interestingly, a number of such circulating miRNAs seem to be promising biomarkers for the major age-related diseases that share a common chronic, low-level proinflammatory status, such as cardiovascular disease (CVD), type 2 diabetes mellitus (T2DM), Alzheimer Disease (AD), rheumatoid arthritis (RA), and cancers.

## Inflamma-miRs

The inflammatory response comprises complex biological reactions that require a fine-tuned integration between a range of immune system cell classes and an extensive network of biomolecules, which until recently had been thought to be largely cytokines. However, identification of a vast repertoire of non-coding microRNA (miRNA) in the mammalian genome has completely revolutionized our understanding of most biological processes, including inflammation (Nilsen, 2007). MiRNAs play a significant role in gene regulation acting as repressors as well as activators, mainly at the post-transcriptional level (Breving and Esquela-Kerscher, 2010). Since a single miRNA can target several genes, and multiple miRNAs share common targets, miRNAs are particularly suited for regulating processes and pathways at the “network” level (Inukai and Slack, 2013).

Sensing of dangerous signals by the innate immune system involves a number of germline-encoded pattern recognition receptors (PRRs) that can detect both conserved pathogen-associated molecular profiles (PAMPs) expressed on micro-organisms and altered endogenous ligands, mostly released by necrotic, senescent, and/or damaged cells (DAMPs). Among PRRs, toll-like receptors (TLRs) play a central role, since their engagement activates a potent proinflammatory pathway (Kawai and Akira, 2011). TLR signaling initiates from different adaptor proteins, such as myeloid differentiation factor 88 (MyD88) or TIR-domain-containing adapter protein-inducing interferon-β, which in turn activate several downstream pathways, leading to activation of transcription factor nuclear factor kappaB (NF-κB), mitogen-activated protein kinases (MAPKs), and members of the interferon regulatory factor family (Dunne and O'Neill, 2005). Fine tuning of TLR signaling prevents generation of harmful and inappropriate inflammatory responses without lowering the surveillance for potentially dangerous signals. Deregulation of the whole network can have destructive effects and lead to tissue damage: this is a hallmark of chronic inflammation, which is often associated with age-related diseases (Olivieri et al., 2013a).

A mounting body of evidence has been documenting a relatively small number of miRNAs that are involved in regulating inflammation: their prototypes are miR-155, miR-21, and miR-146a (Quinn and O'Neill, 2011), hereinafter referred to as *inflamma-miRs*.

In physiological conditions transcription of miR-155, miR-21, and miR-146a is at baseline levels; however, initiation of proinflammatory TLR signaling immediately results in strong co-induction of their expression through a mechanism that is largely NF-κB-dependent (Boldin and Baltimore, 2012).

Although the importance of *inflamma-miRs* in innate immune response regulation is widely accepted, the molecular mechanism of their action has proved to be highly complex. Early investigations disclosed that miR-146a acts as a negative regulator of TLR signaling by targeting both tumor necrosis factor receptor-associated factor 6 (TRAF6) and IL-1 receptor-associated kinase 1 (IRAK-1) (Taganov et al., 2006). MiR-21 was found to down-regulate the expression of IRAK and MyD88 (Chen et al., 2013), as well as of programmed cell death protein 4 (PDCD4), switching the cell program from proinflammatory to anti-inflammatory, mainly as reflected by IL-10 production (Sheddy et al., 2010).

Additional findings showing that miR-155 can also negatively modulate TLR signaling by targeting MyD88, Tak1-binding protein 2 (TAB2) (Ceppi et al., 2009), and the I-κ-B kinase ε (IKKε), (Liang et al., 2011) have generated a model where *inflamma-miRs* operate as a negative feedback loop to protect the organism against overwhelming inflammation. However, subsequent research has disclosed that *inflamma-miRs* could play a dual function, inhibiting as well as inducing TLR signaling (Kondo et al., 2012). This is the case of miR-155, which both suppresses and enhances TLR signaling by silencing Src homology 2 domain-containing protein tyrosine phosphatase-1 (SHP-1), a negative regulator of IRAK activity (O'Connell et al., 2009); suppressor of cytokine signaling 1 (SOCS1); and B-cell lymphoma 6 protein (BCL6), a transcription factor that attenuates NF-κB signaling (Nazari-Jahantigh et al., 2012). A similar action is also exerted by miR-21, which can function as an agonist of single-stranded RNA-binding TLRs and can therefore induce NF-κB activation and secretion of inflammatory molecules (Fabbri et al., 2012).

Although *inflamma-miRs* are co-induced during TLR signaling, several observations suggest that they do not act redundantly and simultaneously, but rather cooperate to control TLR signaling through functionally different performances. At least for miR-146a and miR-155 this has recently been confirmed by Schulte and colleagues, who found different induction behaviors and mRNA target profiles in the response to microbial lipopolysaccaride (LPS), (Schulte et al., 2013). Such coordination of *inflamma-miRs* could go beyond suppression of TLR signaling and contribute to regulating the immunity/inflammation balance, as proposed by Akira's group (Kondo et al., 2012).

Knockout mouse models have been instrumental in shedding light on the role of *inflamma-miRs* in inflammation and inflammation-related diseases. Transcriptome analysis of bic/miR-155-deficient CD4+ T cells in mice identified a wide spectrum of miR-155-regulated genes, including cytokines, chemokines, and transcription factors, suggesting that bic/miR-155 plays a key role in immune system homeostasis and function (Rodriguez et al., 2007). Knockout of miR-146a gene in C57BL/6 mice involves increased transcription of NF-κB-regulated genes (Zhao et al., 2011). These animals also develop myeloid sarcomas and lymphomas as well as chronic myeloproliferation in bone marrow. Genetic ablation of NF-κB p50 suppresses the myeloproliferation, demonstrating that NF-κB dysregulation is responsible for the myeloproliferative disease (Zhao et al., 2011).

Details on the role of *inflamma-miRs* in controlling TLR signaling are just beginning to be explored, and further investigations are warranted to gain insights not only into their individual contribution to the homeostasis of the innate immune response, but also into the consequences of their deregulation in conditions characterized by chronic inflammation.

The most recent evidence for the involvement of *inflamma-miRs* in modulating the proinflammatory response at the cell level is summarized in Table 1. Few cellular *inflamma-miRs* have been reported to be modulated in plasma/serum samples in different physiopathological conditions, as depicted in Table 2.

**Table 1:** **Cellular *inflamma-miRs***.

**MiRs**	**Cell types**	**mRNA targets**	**Signaling**	**References**
MiR-9	Polymorphonuclear neutrophils and monocytes	NFKB1	TLRs	Bazzoni et al., 2009
MiR-21	Monocytes	TLRs	TLRs	Crone et al., 2012
	Myofibroblasts	PDCD4	TLRs	Yao et al., 2011
	HUVEC	PPARα		Zhou et al., 2011
	Immune cells	TLR-8	TLRs	Fabbri et al., 2012
	Hepatocytes	MyD88	TLRs	Chen et al., 2013
	Hepatocytes	IRAK1	TLRs	Chen et al., 2013
MiR-29a	Immune cells	TLR-8	TLRs	Fabbri et al., 2012
MiR-125a	Diffuse large B-cell lymphoma	TNFAIP3	TLRs	Kim et al., 2012
MiR-125b				
MiR-126	Endothelial cells	VCAM-1	Vascular inflammation	Harris et al., 2008
MiR-146a	Intestinal epithelial cells	IRAK-1	TLRs	Chassin et al., 2012
	Astrocytes	IRAK-1	TLRsTLRs	Chassin et al., 2012
	HUVECs	IRAK-1	TLRs	Iyer et al., 2012
	HUVECs	IRAK-1	TGF-β1	Olivieri et al., 2012a,b
	Myofibroblasts	SMAD4		Liu et al., 2012
MiR-155	MSCs	TAB2	iNOS	Xu et al., 2013
	Macrophages	BCL6	TLRs	Nazari-Jahantigh et al., 2012
	Macrophages	SOCS1	TLRs	Sun et al., 2012
MiR-195	Hepatocellular carcinoma	IKKα, TAB3	TLRs	Ding et al., 2013
MiR-199a	Endometrial stromal cells	IKKβ	TLRs	Dai et al., 2012
MiR-517a/c	Cell lines	TNIP1	TLRs	Olarerin-George et al., 2013
Let-7	Primary cultured T cells	TLR-4	IL-13 secretion,	Kumar et al., 2011
Let-7i	Human biliary epithelial cells		TLRs	Chen et al., 2007

*BCL6, B-cell lymphoma 6 protein; HUVECs, human umbilical vein endothelial cells; IKKα and IKKβ, inhibitor of kappa B (IκB) kinase α and β; iNOS, nitric oxide synthase; IRAK-1, interleukin-1 receptor-associated kinase 1; MSCs, Mesenchymal Stem Cell; NFκB1, nuclear factor κB1; PDCD4, programmed cell death 4; MyD88, myeloid differentiation factor 88; PPARα, peroxisome proliferator-activated receptor-α; SMAD4, SMAD family member 4; SOCS1, suppressor of cytokine signaling 1; TAB2, transforming growth factor-β-activated kinase 1 (TAK1)-binding protein 2; TAB3, TAK1-binding protein 3; TLRs, toll-like receptors; TNFAIP3, tumor necrosis factor, alpha-induced protein 3; TNIP1, TNFAIP3 interacting protein 1; VCAM-1, vascular cell adhesion molecule 1*.

**Table 2:** **Circulating *inflamma-miRs* in age-related diseases**.

**Circulating miRs**	**Disease**	**Sample(s)**	**References**
MiR-9	AD	Cerebrospinal fluid	Alexandrov et al., 2012
MiR-21	AMI, CVD	Plasma	Olivieri et al., 2012a,b
	AD	Cerebrospinal fluid	Alexandrov et al., 2012
	Breast cancer	Serum	Mar-Aguilar et al., 2013
MiR-29a	Colorectal liver metastasis	Serum	Wang et al., 2012
MiR-126	T2DM	Plasma	Zampetaki et al., 2010
	Malignant mesothelioma	Plasma	Tomasetti et al., 2012
MiR-146	T2DM	Plasma	Balasubramanyam et al., 2011
	AMI, CVD	Plasma	Olivieri et al., 2012a,b
	AD	Cerebrospinal fluid	Alexandrov et al., 2012
MiR-155	AMI	Plasma	Matsumoto et al., 2012
	Atherosclerosis	Plasma	Wei et al., 2013
	Breast cancer	Serum	Mar-Aguilar et al., 2013
			Liu et al., 2013

*AD, Alzheimer Disease; AMI, acute myocardial infarction; CVD, cardiovascular disease; T2DM, type 2 diabetes mellitus*.

## Relationship between tissue and circulating *Inflamma-miRs*

It is becoming increasingly clear that multiple miRNAs can be deregulated in several diseases (Reid et al., 2011). Detection and identification of stable miRNAs in body fluids has opened a new era in systemic biomarker research directed at improving clinical diagnosis/prognosis within translational medicine. The numerous reports of changes in miRNA expression have often failed to clarify whether they are the cause or the effect of malfunction. Identification of multiple miRNA changes in plasma is important, because specific miRNA combinations unique to a normal physiological or pathological state can provide a useful reference. These findings also raise several questions: do circulating miRNA levels match tissue expression levels? If this is the case, does it also apply to pathological conditions? A greater understanding of the relationship between the level of tissue and plasma miRNAs should help uncover the origin and/or function of circulating miRNAs.

So far only a limited number of studies, mostly in oncology, have addressed the issue. Findings have been controversial: some researchers described a similar trend of alteration both in circulating and tissue miRNAs (Brase et al., 2011), whereas others reported that only a subset of circulating miRNAs reflect tissue cellular abundance (e.g., mammary epithelial tumor), and suggested that cells might have developed a mechanism that selects specific miRNAs for release or retention (Pigati et al., 2010). Studies of animal models found distinct roles for circulating and tissue miRNAs (Waters et al., 2012).

The way circulating miRNAs are delivered into the bloodstream is also poorly understood. Evidence from several studies indicates that miRNAs exist freely in the systemic circulation despite their susceptibility to degradation by extracellular RNAses, raising the issue of which mechanisms underpin their unexpected stability. On the one hand, different studies have revealed that miRNAs are secreted into the extracellular space or the bloodstream either in microvesicles or in exosomes (Valadi et al., 2007; Collino et al., 2010; Hosoda et al., 2011). Secretory exosomes are promising candidates for intercellular miRNAs transfer not only because they provide a protected environment, but also because they may directly transfer internal components to target cells by receptor-mediated interactions. This is consistent with the observation that plasma exosomes can deliver exogenous short interfering RNA to monocytes and lymphocytes (Wahlgren et al., 2012). On the other hand, a number of reports have shown that a significant fraction of extracellular miRNAs reside outside vesicles and act in an exosome-independent manner, protected by RNA-binding proteins such as nucleophosmin 1 (NPM1) (Wang et al., 2010) or argonaute protein 2 (Ago2), (Turchinovich et al., 2011). Whether circulating miRNAs are found in soluble free form or are predominantly transported *via* secreted microvesicles/exosomes is still unclear, since evidence has been found for both mechanisms. However, it has recently been reported that the form of delivery of circulating miRNAs could depend on the type of tissue injury, suggesting a different role for each mode of systemic transport. Bala and co-workers documented this mechanism for circulating *inflamma-miRs* using different liver disease mouse models: in inflammatory liver injury and alcoholic liver disease (ALD) serum/plasma miR-122 and miR-155 were predominantly associated with the exosome-rich fraction, whereas in drug (acetaminophen, APAP)-induced liver injury (DILI/APAP) the same miRNAs were found mainly in protein-rich, soluble free form (Bala et al., 2012).

Evidence has also been found that blood cells are the major contributors to circulating miRNAs (Pritchard et al., 2012), However, analysis of *inflamma-miRs* from patients with acute coronary syndrome (ACS) documented that their levels in plasma and peripheral blood mononuclear cells (PBMCs) did not correlate in all subjects (Yao et al., 2011). This suggests that other cells may contribute to circulating *inflamma-miRs*. Endothelial cells could exert such a role, especially during normal aging and in age related-diseases. Indeed human endothelial cells and circulating progenitor endothelial cells can acquire the senescence-associated secretory phenotype (SASP) during replicative senescence *in vitro* (Olivieri et al., 2012b, 2013c). The accumulation of endothelial senescent cells *in vivo*, and stimulation of immune cells over time, could strongly contribute to promoting and maintaining low-level chronic systemic inflammation (Orjalo et al., 2009; Davalos et al., 2010; Campisi et al., 2011).

## Circulating *Inflamma-miRs* in aging

A progressive increase in circulating acute-phase proteins and proinflammatory mediators, i.e., proteases, cytokines, chemokines, and growth factors, has been described as a general feature of the aging process and has been denominated *inflamm-aging* (Franceschi et al., 2000). Age-associated chronic inflammation has mainly been attributed to progressive activation of immune cells over time and to accumulation of senescent cells with a proinflammatory secretory phenotype (Olivieri et al., 2013b). The complex *inflamma-ging* phenotype is the result of age-related cell/tissue adaptation and remodeling interacting with genetic/epigenetic factors.

Even though TLR family members do not show consistent age-dependent changes across model systems, there is evidence for impaired downstream signaling events during aging, including inhibition of positive effectors and activation of negative modulators of TLR signaling (Olivieri et al., 2013a). Therefore, during aging *inflamma-miR* levels progressively increase in order to stem the cell and tissue damage induced by the low-level chronic inflammation, also likely sustained by the cell senescence secretome (Murray and Smale, 2012).

We have recently described an increased expression of miR-146a in human umbilical vein endothelial cells (HUVECs) and in aortic and coronary endothelial cells (respectively HAECs and HCAECs) during replicative senescence, thus demonstrating that TLR/NF-κB activation and cell senescence can be modulated by the same miRNAs (Olivieri et al., 2012b). MiR-146a is highly expressed also in aged mice (Jiang et al., 2012). In addition, lack of response by aged mouse macrophages to stimulation with LPS and proinflammatory cytokines indicates interruption of the negative feedback loop of miR-146a (Jiang et al., 2012). Altogether, these data lend support to the hypothesis that cellular senescence and TLR signaling activation may be closely interconnected and share common regulators.

Given the involvement of miRNAs in gene expression regulation, a peculiar modulation of their expression might contribute to efficient homeostasis in human aging. It is worth stressing that exceptionally long survival requires dynamic preservation of optimal levels of physiological variables, and that the mean levels of many biomarkers of aging are not stable, but change in the course of life (Spazzafumo et al., 2013). Only four studies have compared the miRNA expression profile of centenarians and younger subjects. They have shown a significant overlap between the miRNA profiles of centenarians and young individuals and a different profile in octogenarians, supporting the hypothesis that achievement of extreme longevity probably requires a special gene expression regulation (ElSharawy et al., 2012; Gombar et al., 2012; Olivieri et al., 2012a; Serna et al., 2012). Interestingly, all these studies showed miR-21 deregulation in centenarians compared with younger subjects. Age-related changes in the expression of miR-21 and miR-21^*^ have recently been reported also in mouse heart, the major changes occurring from middle to old age (Zhang et al., 2012).

Modulation of miR-21 expression in plasma, circulating cells, and tissues of very old subjects and animals is not surprising to those who believe that miR-21 lies at the intersection of senescence, inflammation, and age-related diseases. Our group showed a positive correlations between circulating miR-21 and two important biomarkers of inflammation: C-reactive protein (CRP) and fibrinogen (Olivieri et al., 2012a,b). These data suggest that centenarians may have a better balance of their systemic inflammatory status compared with elderly subjects. Notably, we also found age-related changes in circulating miR-146a levels that were quite similar to those described for miR-21 (Olivieri et al., 2012a,b, our unpublished data). Interestingly, analysis of global miRNA expression in the peripheral blood of adult women has shown that miR-155 is one of the most up-regulated miRNAs among older women (Sredni et al., 2011).

## Circulating *Inflamma-miRs* in age-related diseases

Abundant data continue to support the hypothesis that progressive up-regulation of inflammatory gene expression and high levels of inflammatory signaling facilitate the development and progression of the major age-related diseases, such as cardiovascular disease (CVD), type 2 diabetes mellitus (T2DM), Alzheimer Disease (AD), rheumatoid arthritis (RA), and cancers. Patients suffering from such diseases show subclinical/clinical inflammation and, interestingly, deregulation of most circulating *inflamma-miRs* (Wang et al., 2012). Since multiple co-expressed miRNAs can cooperatively regulate a given biological process by targeting common components of that process, the development and progression of human diseases could be associated with abnormal regulation of multiple miRNAs functioning cooperatively. Some of the most recent data showing that cellular and circulating *inflamma-miR* deregulation is shared by the major human age-related diseases are summarized below.

### *Inflamma-miRs* and T2DM

Recently, some of the miRNAs that we have designated *inflamma-miR*s, including miR-21 and miR-146a, were identified as novel players in β-cell failure elicited by proinflammatory cytokines in both *in vitro* and *in vivo* animal models (Roggli et al., 2010). Reduced miR-146a levels were also reported in PBMCs from Asian Indian patients with T2DM, in association with insulin resistance, poor glycaemia control, specific proinflammatory cytokine gene variants, and high levels of plasma TNFα and IL-6 (Balasubramanyam et al., 2011). Importantly, glucose concentrations were also reported to correlate negatively with circulating miR-126 in endothelial apoptotic bodies (Zampetaki et al., 2010); similarly, in patients with diabetes the reduction in miR-126 was seen to be confined to circulating vesicles in plasma (Zampetaki et al., 2010). Interestingly, miR-126 can modulate endothelial cell activation in response to systemic inflammatory stimuli, and can down-regulate the expression of IKBα, an important inhibitor of NF-κB signaling (Asgeirsdóttir et al., 2012; Feng et al., 2012).

### *Inflamma-miRs* and CVD

In patients with CVD activation of innate immunity leads to an acute inflammatory reaction. There is evidence that *inflamma-miR* may contribute to the development/restraint of this inflammatory response: circulating *inflamma-miRs* could thus be clinically relevant diagnostic/prognostic biomarkers in CVD patients. MiR-21 plays important roles in cardiovascular and pulmonary disorders, including cardiac and pulmonary fibrosis and myocardial infarction, and also regulates various immunological and developmental processes (Kumarswamy et al., 2011).

We recently reported significantly higher circulating miR-21 and miR-146a levels in elderly patients with acute myocardial infarction (AMI) and/or heart failure compared with healthy subjects (Olivieri et al., 2012c). Circulating miR-155 were higher in those post-AMI patients who experienced cardiac death within 1 year (Matsumoto et al., 2012). Elevated miR-155 levels are also found in proinflammatory macrophages and atherosclerotic lesions, even though the effects of miR-155 seem to be different in early vs. advanced atherosclerosis (Wei et al., 2013). Recent studies suggest that miRNA deregulation may limit cardiovascular repair responses and result in an altered function and differentiation of cardiovascular progenitor cells and endothelial progenitor cells (EPCs), modulating endothelial regeneration and cardiomyocyte homeostasis and playing a crucial role in CVD (Jakob and Landmesser, 2012). Aging-associated senescence results in reduced EPC number and function, contributing to enhanced cardiac risk, reduced angiogenic capacity, and impaired cardiac repair effectiveness. Mounting evidence supports a role for miRNAs in vascular homeostasis, and miR-21 was found to regulate EPC senescence (Zhu et al., 2013).

Overall, circulating miR-146a, miR-155, and miR-21 are up-regulated in plasma of CVD patients.

### *Inflamma-miRs* and AD

Increasing evidence supports a major role for TLRs in brain injury and their involvement in neurodegenerative disorders including AD. Components of this inflammatory pathway are known to contribute to AD, in part through overexpression of IL-1α and promotion of 42-amino acid amyloid β 42 (Aβ42) peptide generation; in turn IL-1α and amyloid β induce transcription of the proinflammatory prostaglandin synthase cyclooxygenase-2 (COX-2) gene and stimulate apoptotic brain cell death and neural tissue degeneration (Mrak and Griffin, 2001). Consistent with these findings many *inflamma-miRs*, including miR-9, miR-146a, and miR-155, are strongly expressed in human cerebrospinal fluid (CSF)- and brain tissue-derived extracellular fluid (ECF) from AD patients and are significantly up-regulated compared with age-matched controls, suggesting that they may be involved in modulation or promotion of miRNA-triggered pathogenic signaling throughout the brain and the CNS (Alexandrov et al., 2012). In AD patients miR-125b, miR-146a, and miR-155 have been shown to down-regulate complement Factor H (CFH), an important repressor of innate immunity acting on the cerebral inflammation response (Lukiw and Alexandrov, 2012; Lukiw et al., 2012a).

MiR-146a is among the more extensively investigated miRNAs in AD: it is up-regulated in response to IL-1, Aβ42 and oxidative stress in cultured human neuronal glial cells (Lukiw et al., 2008; Cui et al., 2010; Holohan et al., 2012; Lukiw and Alexandrov, 2012). In line with these data it is over-expressed in temporal cortices and in Aβ42-stressed human astroglial cells, where it down-regulates IRAK1 while inducing compensatory up-regulation of IRAK2 (Cui et al., 2010).

Interestingly, miR-146a and miR-155 were detected in the secretion of stressed human primary neural cells, and the conditioned medium containing miR-146a and miR-155 was found to induce Alzheimer-type gene expression changes in control brain cells (Lukiw et al., 2012b). These data suggest that paracrine transfer of genetic information between cells—either within the local brain environment or in the cerebrospinal or systemic circulation—may be the source of both beneficial and detrimental signals that further modulate the amyloidogenic, inflammatory, or neurotrophic aspects of the AD process (Lukiw, 2012).

Further confirmation that inflammatory pathways can contribute to AD development comes from the recent identification of a role for triggering receptor expressed on myeloid cells 2 (*TREM2)* in modulating the risk of AD onset (Guerreiro et al., 2013; Jonsson et al., 2013). TREM2 is an innate immune receptor expressed on the cell surface of microglia, macrophages, immature dendritic cells, and white matter in the hippocampus and neocortex, two areas that partially overlap with those affected by AD (Jiang et al., 2013). It controls two signaling pathways, one enhancing phagocytosis and another suppressing inflammatory reactivity. Reduced *TREM2* expression is associated with an increase in microgliosis and neurodegeneration, suggesting that it modulates AD by enhancing inflammation (Jiang et al., 2013).

Although the analysis of miRNAs in AD is a relatively new research area, the data reported so far strongly indicate that *inflamma-miRs* could play a key role also in this condition.

## *Inflamma-miRs* and RA

Altered miRNA expression has been demonstrated in the inflamed joints of RA patients (Blüml et al., 2011). Interestingly, miR-155 up-regulation has been documented in synovial membrane and synovial fluid (SF) macrophages from RA patients in association with reduced expression of its target, SHIP-1, an inhibitor of inflammation (Kurowska-Stolarska et al., 2011). Moreover miR-155(−/−) mice show significantly reduced local bone destruction, attributed to reduced osteoclast generation, although the severity of the joint inflammation is similar to the one seen in wild-type mice. These data demonstrate that miR-155 is critically involved in the adaptive and innate immune reactions leading to autoimmune arthritis (Blüml et al., 2011). In addition up-regulation of miR-146a, detected in PBMCs from RA patients, suggests that this miRNA could be a useful marker of disease activity (Abou-Zeid et al., 2011; Xie et al., 2013). Interestingly, circulating miR-21 is increased in the blood of patients with systemic lupus erythematosus (SLE) or RA, whereas circulating miR-146a and miR-155 show a trend toward significantly reduced levels only in SLE (Carlsen et al., 2013).

### *Inflamma-miRs* and cancers

MiRNAs are increasingly being recognized as oncogenes or oncosuppressors, since they contribute to cell transformation. Solid tumors are infiltrated by different stromal cells, e.g., fibroblasts, endothelial cells, lymphocytes and macrophages, that strongly influence neoplastic processes through a continuous heterotypic cross-talk (Squadrito et al., 2013). It is therefore not surprising that *inflamma-miRs* have been recognized as modulators of cancer development and progression (Williams et al., 2008). The contribution of miR-146a deregulation has been widely documented (Hurst et al., 2009; Li et al., 2010; Labbaye and Testa, 2012). However, its mechanism of action remains elusive, since both raised and decreased levels have been described depending on the type of cancer (Williams et al., 2008). Since miR-146a acts as a negative feedback loop of inflammation, dynamic changes in its expression can be expected in cancer tissues depending on the context of the heterotypic cross-talk.

Interestingly, mice with miR-146a deletion spontaneously develop subcutaneous flank tumors (Zhao et al., 2011). MiR-146a has been reported to suppress metastatic activity (Hou et al., 2012; Hwang et al., 2012), in particular its up-regulation inhibits cancer cell invasion and metastasis *in vitro* and *in vivo* (Hou et al., 2012). Altogether these findings show that by counteracting the inflammatory state associated with cell senescence, miR-146a can exert a general tumor suppressing action by inhibiting cancer development and cancer cell invasion and metastasis.

MiR-21 is frequently up-regulated in cancer and is implicated in practically every stage of the cancer process: promotion of cell proliferation, invasion, and metastasis; genome instability and mutation; inflammation; replicative immortalization;abnormal metabolism; angiogenesis; evasion of apoptosis; immune destruction and growth suppression. This suggests that miR-21 is an oncogene with a key role in resisting programmed cell death in cancer cells (Buscaglia and Li, 2011). MiR-155 is also an established “*oncomiR”* in breast cancer and regulates several pro-oncogenic pathways including angiogenesis (Czyzyk-Krzeska and Zhang, 2013).

## Conclusion

Recent data on inflammation-related miRNAs in normal and pathological aging outline a complex scenario characterized by an altered expression of specific miRNAs that we have named *inflamma-miRs*, which mainly target the TLRs/NF-κB pathway. Up-regulation of inflammation markers is a general feature of the aging process and has been named *inflamm-aging*. The *inflamm-aging* phenotype results from age-related cell and tissue adaptation/remodeling interacting with the genetic/epigenetic background. It is a complex phenotype involving not only innate but also adaptive immunity and affecting a range of tissues and organs such as gut, fat, liver, muscle and brain. Importantly, *inflamm-aging* appears to be accelerated in a variety of age-associated diseases. Tissue and circulating *inflamma-miRs* could contribute to restrain the activity of the senescent cell secretome and to check the destruction induced by activation of the inflammatory response (Murray and Smale, 2012; Olivieri et al., 2013a). *Inflamma-miRs* have been implicated in regulation of the immune and inflammatory response, and their abnormal expression may contribute to the low-level chronic inflammation that has been documented both in normal aging and in the major age-related diseases. Circulating *inflamma-miRs* could thus have diagnostic/prognostic relevance in human diseases, e.g., CVD, T2DM, AD, RA, and cancer, which share a common inflammatory background. Recent data show up-regulation of *inflamma-miRs* in the circulation of healthy elderly and old individuals: the increase is less pronounced in centenarians and greater in patients with CVD, AD, or cancer. Notably, T2DM patients show decreased levels of circulating *inflamma-miRs*, but these data need to be confirmed in patients with diabetic complications. It is conceivable that the main sources of circulating *inflamma-miRs* in aging and age-related diseases are immunity circulating/tissue cells and endothelial circulating/resident cells. Cell senescence and inflammatory stimulation can contribute to induce and perpetuate systemic inflammation over time, inducing up-regulation of *inflamma-miRs* to stem the excessive activation of inflammatory pathways.

Since this field of research is new and growing, it would not be surprising if in the near future novel miRNAs were recognized as fine tuners of inflammation and thus added to the list of *inflamma-miRs*.

### Conflict of interest statement

The authors declare that the research was conducted in the absence of any commercial or financial relationships that could be construed as a potential conflict of interest.
